# Melatonin promotes sirtuin 1 expression and inhibits IRE1α–XBP1S–CHOP to reduce endoplasmic reticulum stress–mediated apoptosis in chondrocytes

**DOI:** 10.3389/fphar.2022.940629

**Published:** 2022-08-11

**Authors:** Kunpeng Qin, Hao Tang, Yi Ren, Di Yang, Yetian Li, Wei Huang, Yunfeng Wu, Zongsheng Yin

**Affiliations:** ^1^ Department of Orthopaedics, The First Affiliated Hospital of Anhui Medical University, Hefei, China; ^2^ The Key Laboratory of Microbiology and Parasitology of Anhui Province, The Key Laboratory of Zoonoses of High Institutions in Anhui, Anhui Medical University, Hefei, China; ^3^ Department of Orthopaedics, The First Affiliated Hospital of USTC, Division of Life Sciences and Medicine, University of Science and Technology of China, Hefei, China; ^4^ Department of Orthopaedics, The First Affiliated Hospital of Anhui Medical University, Anhui China. Anhui Public Health Clinical Center, Hefei, China

**Keywords:** melatonin, osteoarthritis, endoplasmic reticulum stress, apoptosis, IRE1α--XBP1S--CHOP

## Abstract

Osteoarthritis (OA) is the most common chronic disease characterized by a loss of chondrocytes and the degeneration of cartilage. Inflammation plays an important role in the pathogenesis and progression of OA via the activation of the endoplasmic reticulum (ER) stress signaling pathway. In this study, we stimulated human primary chondrocytes with lipopolysaccharide (LPS) to reduce cell viability and induce chondrocyte apoptosis. LPS–stimulated human primary chondrocytes induced ER stress and significantly upregulated the ER chaperone glucose–regulated protein 78 (GRP78) and increased the expression level of C/EBP–homologous protein (CHOP), a key mediator of ER stress––induced apoptosis. Interestingly, melatonin treatment attenuated ER stress–mediated chondrocyte apoptosis. Melatonin inhibited the expression of cleaved caspase-3, cleaved caspase-10, Bax, CHOP, GRP78, cleaved caspase-4, phospho–inositol–requiring enzyme 1α (P-IRE1α), and spliced X-box-binding protein 1 (XBP1S). In an anterior cruciate ligament transection mouse model of OA, melatonin (50 and 150 mg/kg) dose–dependently relieved joint cartilage degeneration and inhibitied of chondrocyte apoptosis. Immunohistochemical analysis indicated that melatonin could promote SIRT1 the expression and inhibit CHOP and cleaved caspase-3 expression in OA mice. In conclusion, our findings demonstrate for the first time that melatonin inhibits the IRE1α-XBP1S-CHOP signaling pathway by promoting the expression of SIRT1 in LPS-treated human chondrocytes and delaying OA progression *in vivo*.

## Introduction

Osteoarthritis (OA) is a common degenerative condition that affects many people worldwide. Its primary causes include obesity, aging, genetics, and trauma, and it is characterized by a loss of cartilage cells and the gradual degradation of cartilage (Li et al., 2014; Uehara et al., 2014; Chen et al., 2017; Shi et al., 2017). Chondrocytes, the only cells in articular cartilage, are primarily responsible for maintaining the dynamic balance of articular cartilage by regulating a anabolism and catabolism of the extracellular matrix (Thomas et al., 2007). Persistent inflammation and apoptosis of chondrocytes are important processes involved in the development of OA (Kaczanowski, 2016; Moon et al., 2016; Robinson et al., 2016; Dai et al., 2018). Apoptosis is a process that is regulated by genes and ultimately leads to cell death (D’arcy, 2019). Currently there are three known triggers of apoptosis, the exogenous apoptosis pathway, the mitochondrial–mediated endogenous apoptosis pathway, and the endoplasmic reticulum (ER) stress (ERS)–mediated apoptosis pathway. Of these, the ERS-mediated apoptosis pathway has attracted the most attention (Kaczanowski, 2016; Moon et al., 2016).

Accumulation of misfolded proteins within the ER lumen leads to ER stress. Stressed cells then activate a downstream adaptive mechanism to alleviate the stress and restore ER homeostasis (Hetz et al., 2020). This adaptive mechanism is known as the unfolded protein response (UPR) (Tavernier et al., 2017). Mild ERS helps misfolded and unfolded proteins to fold correctly, and supports the degradation of misfolded proteins, thereby promoting cell survival and maintaining balance in the intracellular environment. If stress factors persist however, ERS will exceed the UPR threshold and cell apoptosis will be induced (Xin et al., 2014; Sepulveda et al., 2018). UPR occurs mainly through the activation of resident ER, activating transcription factor 6 (ATF6), protein kinase R (PRK)-like ER kinase (PERK), and inositol-requiring protein 1-α (IRE1-α)-sensor transmembrane proteins by activating the corresponding signaling pathways (Abdullah and Ravanan, 2018). PERK-eukaryotic initiation factor 2α (eIF2α)-C/EBP homologous protein (CHOP), IRE1α-X-box-binding protein 1 (XBP1)-CHOP, and ATF6-XBP1-CHOP have been described as the three pathways key to inducing apoptosis (Komoike and Matsuoka, 2016). As the most conservative signaling pathway in the UPR, the IRE1α-XBP1-CHOP pathway plays a significant role in ERS and is considered a promising target for drug therapy. The IRE1α signaling pathway and its relationship with chondrocyte apoptosis have been extensively studied (Wu et al., 2018). Inhibition of apoptosis and the promotion of cartilage regeneration are both of clinical significance with respect to delaying the development of OA by directly blocking the IRE1α pathway. To date however, no relevant *in vivo* or *in vitro* studies have been published (Huang et al., 2021). A sustained inflammatory response during OA results in chronic ER stress (Zhang et al., 2019a); thus, in the current study lipopolysaccharide (LPS) was used to induce ERS and apoptosis to investigate OA treatment mechanisms.

Accumulation of misfolded proteins within the ER lumen leads to ER stress. Stressed cells then activate a downstream adaptive mechanism to alleviate the stress and restore ER homeostasis (Hetz et al., 2020). This adaptive mechanism is known as the unfolded protein response (UPR) (Tavernier et al., 2017). Mild ERS helps misfolded and unfolded proteins to fold correctly, and supports the degradation of misfolded proteins, thereby promoting cell survival and maintaining balance in the intracellular environment. If stress factors persist however, ERS will exceed the UPR threshold and cell apoptosis will be induced (Xin et al., 2014, Sepulveda et al., 2018). UPR occurs mainly through the activation of resident ER, activating transcription factor 6 (ATF6), protein kinase R (PRK)-like ER kinase (PERK), and inositol&hyphen;requiring protein 1-α (IRE1-α)-sensor transmembrane proteins by activating the corresponding signaling pathways (Abdullah and Ravanan, 2018). PERK-eukaryotic initiation factor 2α (eIF2α)-C/EBP homologous protein (CHOP), IRE1α-X-box-binding protein 1 (XBP1)-CHOP, and ATF6-XBP1-CHOP have been described as the three pathways key to inducing apoptosis (Komoike and Matsuoka, 2016). As the most conservative signaling pathway in the UPR, the IRE1α-XBP1-CHOP pathway plays a significant role in ERS and is considered a promising target for drug therapy. The IRE1α signaling pathway and its relationship with chondrocyte apoptosis have been extensively studied (Wu et al., 2018). Inhibition of apoptosis and the promotion of cartilage regeneration are both of clinical significance with respect to delaying the development of OA by directly blocking the IRE1α pathway. To date however, no relevant *in vivo* or *in vitro* studies have been published (Huang et al., 2021). A sustained inflammatory response during OA results in chronic ER stress (Zhang et al., 2019a); thus, in the current study lipopolysaccharide (LPS) was used to induce ERS and apoptosis to investigate OA treatment mechanisms.

Silent information regulator 2 type 1 (SIRT1) has been implicated in several age-related conditions such as cancer, obesity, cardiovascular disease, dementia, type 2 diabetes, arthritis, osteoporosis, and OA (Morris, 2013). It is a histone deacetylase that relies on nicotinamide adenine dinucleotide, which has a protective effect in human chondrocytes and can prevent OA progression (Lim et al., 2012; Terauchi et al., 2016). It can reportedly promote cell survival while suppressing cell apoptosis by modulating multiple transcription factors, including nuclear factor-κB, p53, forkhead box O protein, DNA repair factor Ku70, and transcription co-activator p300 (Vaziri et al., 2001; Brunet et al., 2004; Cohen et al., 2004; Yeung et al., 2004; Bouras et al., 2005). The protective role of SIRT1 in ERS-induced cells has been demonstrated in previous investigations (Feng et al., 2019; Luo et al., 2019; Hu et al., 2020). Previous studies have also shown that SIRT1 levels in normal cartilage samples taken from elderly individuals are lower than those in samples taken from younger people, and that SIRT1 levels in samples from OA patients are lower than those in samples from healthy people. SIRT1 is a potential therapeutic option for the treatment of OA because it plays a crucial role in articular cartilage protection (Sacitharan et al., 2020). Luo et al. (Luo et al., 2019) reported that SIRT1 reduces hypoxia-induced apoptosis through the IRE1α pathway, thereby protecting cardiomyocytes from hypoxic stress. It was recently shown that Sirt1 inhibition induces hyperacetylation and phosphorylation of eIF2α and PERK to regulate PERK-ATF4 signaling of ER stress (Prola et al., 2017; Kang et al., 2018). Additionally, SIRT1 deacetylates PERK physically through physical interactions (Zhang et al., 2021). It has been shown that SIRT1 deacetylates XBP1s and inhibits the transcriptional activity of XBP1s to regulate UPR signaling (Wang et al., 2011). Luo and collaborators reported that reported that SIRT1 reduces hypoxia–induced apoptosis through the IRE1α pathway, thereby protecting cardiomyocytes from hypoxic stress. (Luo et al., 2019). However, whether SIRT1 can regulate ERS and slow the progression of OA in human chondrocytes via the IRE1α-XBP1S-CHOP pathway is unclear.

N-acetyl-5-methoxy tryptamine (melatonin) is a hormone produced by the pineal gland in mammals, including humans. It has a variety of biological functions including anti-oxidative, anti-inflammatory, and anti-apoptotic effects (Mazzon et al., 2006; García et al., 2014). It is evidently effective for the treatment of several pathological conditions including cancer, neurotoxicity, OA, and liver and metabolic diseases, but the specific mechanisms by which melatonin exerts its effects remain unclear (Pei et al., 2009; Liu et al., 2014). Many *in vivo* and *in vitro* studies have shown that melatonin inhibits ERS-mediated apoptosis in some cells (Chen Y. et al., 2016; Zhou et al., 2020; Qin et al., 2021). In a recent study melatonin prevented chronic obstructive pulmonary disease by inhibiting ERS and apoptosis via the upregulation of SIRT1 expression in mice (He et al., 2019).

However, whether melatonin inhibits ERS-induced apoptosis in human chondrocytes by activating SIRT1 protein levels remains unclear. In the current study the effects of melatonin on LPS-induced chondrocyte apoptosis were investigated, as were the potential mechanisms involved. The therapeutic effects of melatonin were also investigated in a murine model of arthritis of the knee.

## Materials and methods

### Patients and tissue samples

The study was approved by the Research Ethics Committee of the First Affiliated Hospital of Anhui Medical University, china written informed consent was obtained from all individuals before their operations. International Cartilage Repair Society grade 4 human OA cartilage was obtained from the knee joints of 26 OA patients (mean age 62.73 ± 5.5 years) at the First Affiliated Hospital of Anhui Medical University. The cartilage was stored at 4°C for 2–3 h to extract primary cartilage cells.

### Reagents and antibodies

Fetal bovine serum, Dulbecco’s modified Eagle medium (DMEM)/F12 medium, and phosphate-buffered saline (PBS) were purchased from HyClone (Logan, UT, United States). Melatonin (73314) was purchased from Med Chem Express (Princeton, NJ, United States). LPS (ST1470), tunicamycin (TM) (SC0393), 4′,6-diamidino-2-phenylindole (DAPI) (P0131), 0.25% trypsin (C0205), and EX527 were purchased from Beyotime (Shanghai, China). Primary antibodies against SIRT1 (13161-1-AP), Bax (50599–2–Ig), glucose-regulated protein 78 (GRP78) (11587-1-AP), CHOP (15204-1-AP), IRE1α (27528-1-AP), and β-actin (15204-1-p) were purchased from Proteintech (Wuhan, Hubei), as were horseradish peroxidase (HRP)-conjugated secondary antibodies. Phospho-IRE1α (P-IRE1α) (ab124945), Bcl-2 antibodies (ab32124), cleaved caspase-4 (ab22687), and cleaved caspase-10 (ab11475) were purchased from Abcam (Cambridge, United Kingdom), and XBP1S (#40435) and cleaved caspase-3 (Asp175) were purchased from Cell Signaling Technology (Danvers, MA, United States). Cell-counting kit 8 (CCK-8) was purchased from Beyotime.

### Chondrocyte isolation and culture identification and treatment

Primary human knee chondrocytes were isolated from OA cartilage tissue using a previously described protocol (Tardif et al., 2009). Briefly, OA cartilage tissue was collected from the proximal tibia and distal femur and washed three times with sterile PBS. The samples were then sliced into pieces measuring 1 mm^3^, trypsinized in sequential digestion (ethylenediaminetetraacetic acid [EDTA]-free trypsin) for 30 min, and treated with 0.2% type II collagenase dissolved in DMEM/F12 at 37°C for 10 h. Tissues and cells were then passed through a 50-ml filter to remove undigested contents, then cells were transferred into a 15-ml tube and centrifuged. Chondrocytes were then obtained via centrifugation. The supernatant was discarded and cell pellets were resuspended in a 25 cm^2^ cell culture flask with 3 ml of DMEM containing 10% fetal bovine serum and 1% penicillin/streptomycin. Cells were cultured at 37°C in a 5% CO_2_ humified atmosphere. The culture medium was replaced every 2–3 days. After two or three passages, chondrocytes were used in assays. For LPS treatment different concentrations were added to the culture medium (0, 1, 5, 10, 20, or 50 μg/ml), and 10 μg/ml was ultimately chosen for further experimentation. When the cells reached 70–80% confluence the medium was replaced, and the cells were divided into different treatment groups. Melatonin was dissolved in absolute ethyl alcohol to obtain an initial stock concentration of 250 mM, which was then diluted with basic medium to create stocks of 5, 10, 50, and 100 µM for use. There were no concerns of potential absolute ethyl alcohol-mediated toxicity. Chondrocyte identification was performed via Alcian blue staining as previously described (Schofield et al., 1975). In short, chondrocytes were fixed with 4% paraformaldehyde for 20 min, followed by staining with 0.5% Alcian blue for 30 min. They were then washed with distilled water, and assessed via microscopy (LEICA, Wetzlar, Germany).

### Cell viability assay

The viability of articular chondrocytes was assessed using the CCK-8. Chondrocytes were seeded at 5 × 10^4^ cells per well in a 96-well plate well overnight. A drug-inoculation assay was performed when the cells reached 60–70% confluence. Chondrocytes were treated with increasing concentrations of melatonin (0, 5, 10, 50, and 100 µM) or LPS (10 μg/ml) for 24 h, then 100 μl of CCK-8 solution (10 μL CCK-8 + 90 μl basal medium) was added to each well followed by incubation at 37°C in the dark for 2 h. Lastly, the absorbance of each sample was determined via a Thermomax microplate reader (Bio-Tek Instruments, Winooski, VT, United States) at a wavelength of 450 nm. Each sample was plated in triplicate, and data are representative of three independent experiments.

### Flow cytometry

Briefly, chondrocytes in 6-well plates were exposed to LPS, TM (1 μM), a classic ERS inducer, or melatonin + LPS for 24 h. Each group was prepared separately. At the end of the experiment chondrocytes were digested with EDTA-free trypsin and centrifuged to obtain cell pellets. The cell pellets were gently resuspended in 500 μl of 1 × annexin V binding buffer (Beyotime), then stained with annexin V-fluorescein isothiocyanate and propidium iodide for 10 min at room temperature in the dark. The stained chondrocytes were assessed via flow cytometry (BD, Franklin Lakes, NJ, United States), and the rate of apoptosis was expressed as the percentage of cells with annexin V-fluorescein isothiocyanate positivity and propidium iodide positivity/negativity. Each sample was assessed in triplicate, and data are representative of three independent experiments.

### Quantitative real–time polymerase chain reaction

Total RNA was obtained from chondrocytes using TRIzol (Invitrogen, Carlsbad, CA, United States), then 1 μg of RNA was reverse-transcribed into complementary DNA using a specific reverse-transcription kit (Accurate Biotechnology, Changsha, China) in accordance with the manufacturer’s instructions. qPCR Master Mix (Accurate Biotechnology) was then used to perform quantitative real-time polymerase chain reaction (qRT-PCR) assays using corresponding primers. Gene expression levels were normalized to glyceraldehyde 3-phosphate dehydrogenase (GAPDH) levels, and data were quantified via the −ΔΔCt method. Each sample was assessed in triplicate, and data are representative of three independent experiments. The primer sequences used are shown in Table 1.

**TABLE 1:** Primer sequences of target genes.

Gene	Forward primer (5–3′)	Reverse primer (5–3′)
GRP78	AGGGCAACCGCATCACG	ATCGCCAATCAGACG
CHOP	GAA​CAG​TGG​GCA​TCA​CCT​C	CAGTCCCCTCCTCAGCAT
XBP1S	AGCAGCAAGTGGTGGATT	CTCTGGAACCTCGTCA
BCL2	ACG​GTG​GTG​GAG​GAA​CTC​TTC​AG	GGT​GTG​CAG​ATG​CCG​GTT​CAG
SIRT1	GACGACGAGGGCGAGGAG	ACA​GGA​GGT​TGT​CTC​GGT​AGC
COLII	ACG​CTC​AAG​TCG​CTG​AAC​AAC​C	ATC​CAG​TAG​TCT​CCG​CTC​TTC​CAC
GAPDH	TGG​CCT​TCC​GTG​TTC​CTA​C	GAG​TTG​CTG​TTG​AAG​TCG​CA

### Western blot assay

Melatonin-pretreated chondrocytes were incubated with LPS in a 6-well plate. After 24 h chondrocytes were washed with cold PBS and lysed in RIPA lysis buffer containing a protease inhibitor mixture and a phosphatase inhibitor cocktail (Roche Diagnostics, Basel, Switzerland) for 30 min to extract total intracellular proteins. A BCA protein assay kit (Beyotime) was used to measure total protein concentrations. Equal concentrations of total protein were separated on 10% SDS-PAGE gels and transferred to polyvinylidene difluoride membranes (Millipore, Burlington, MA, United States), which were activated by methyl alcohol and incubated overnight at 4°C with primary antibodies against Bcl-2 (1:1,000), cleaved caspase-3 (1:500), Bax (1:2000), SIRT1 (1:500), CHOP (1:500), GRP78 (1:1,000), XBP1S (1:500), IRE1α (1:2000), and P*-*IRE1α (1:1,000). The membranes were then washed for 10 min three times with tris-buffered saline containing 0.1% Tween-20, then incubated with HRP-conjugated secondary antibody for 1 h at room temperature. Images of blots were obtained via the Chemo Dox XRS system (Bio-Rad Laboratories, Hercules, CA, United States). ImageJ version 6.0 (U.S. National Institutes of Health, Bethesda, MD, United States) was used to calculate the optical density of each band. Each sample was assessed in triplicate, and data are representative of three independent experiments.

### Immunofluorescence assays

Chondrocytes were seeded on a slide in a 6-well plate (1 × 10^6^ cells/well) and fixed with freshly prepared 4% paraformaldehyde for 15 min. The cells were then treated with 0.5% (v/v) Triton X-100 for 10 min after being washed three times with PBS and blocked with 5% (w/v) bovine serum albumin for 1 h at room temperature. They were then incubated with primary antibodies against cleaved caspase-3 (1:200), SIRT1 (1:200), CHOP (1:200), and XBP1S (1:500) overnight at 4°C, followed by incubation with fluorescent secondary antibodies (1:300) (Thermo Fisher Scientific, Waltham, MA, United States) in a dark room. DAPI (Beyotime) was subsequently added for 2 min. An inverted fluorescence microscope (LEICA) was used to examine and photograph the samples. Each sample was assessed in triplicate, and data are representative of three independent experiments.

### Transmission electron microscopy

Human chondrocytes were cultured in 25 cm^2^ culture flasks. We washed them with PBS and collected cells with 0.05% trypsin–free EDTA treatment. The cell aggregates were fixed with 2.5% glutaraldehyde, and the samples were dehydrated by a series of incubations in 50, 70, 90, and 100% ethanol, then dehydrated with 100% acetone and embedded in epoxy resin. An ultramicrotome was used to cut the fixed cell aggregates into ultrathin sections, stained them, and inspected their ultrastructure using a transmission electron microscope (Talos L120C G2; Thermo Fisher Scientific, Waltham, MA, United States).

### Mouse OA model

All mouse experiments were approved by the Ethics Committee for Animal Research, Anhui Medical University, Anhui, China. Forty 10–12 week-old male C57/BL mice weighing 25–30 g were provided by the Laboratory Animal Center of Anhui Medical University (He Fei, China). OA was induced in the mice via anterior cruciate ligament (ACL) transection (ACLT) of the right knee. Arthrotomy without transection of the ACL in the right knee joint was also performed in 10 C57/BL mice, which were used as a control group. The mice were randomly divided into four groups: a sham group, an ACLT group, an ACLT + low-dose melatonin treatment group, and an ACLT + high-dose melatonin treatment group. After the ACLT surgery, melatonin was injected intraperitoneally once a day for 8 weeks. Briefly, anesthesia was induced via intraperitoneal injection of 50 mg/L chloral hydrate, hair was shaved at the operation site, and the left posterior region was fixed in the supine position. The knee joint was exposed after a medial capsular incision and gentle lateral displacement of the extensor mechanism without transection of the patellar ligament, the ACL was transected, and the joints were flushed with sterile saline prior to closure of the joint capsule. The articular cavity was then sutured with 7–0 surgical sutures, and the skin wound was closed. Topical amoxicillin was applied to prevent wound infection. Anesthesia recovery and wound healing were monitored.

### Intraperitoneal injection of melatonin

Melatonin (100 mg) was dissolved in 1 ml of absolute ethanol then diluted to a final concentration of 10 mg/ml in normal saline. Mice were intraperitoneally injected with either low-dose melatonin (50 mg/kg) or high-dose melatonin (150 mg/kg) once a day for 8 weeks (Zhang et al., 2019b). They were then euthanized, and knee joint samples were collected.

### Micro-computed tomography

Knee joints were evaluated via micro-computed tomography (CT) (Sky Scan 1,176; Bruker, Billerica, MA, United States), conducted for 120 min at 800 μA and 50 kV with a resolution of 12 μm. Micro-CT data were analyzed by CTAn (SkyScan, United States) and mimics medical 21 (Materialise, Belgium). The 3D structural parameters analyzed included the BMD and region of interest of the subchondral region of the tibial plateau, selected for analysis with the following morphological parameters: 1) Bone volume/total tissue volume (BV/TV) (%); 2) trabecular thickness (Tb.Th) (mm); 3) trabecular number (Tb.N) (1/mm); 4) trabecular separation (Tb.Sp) (mm); and 5) trabecular mesh factor (Tb.Pf) (1/mm).

### Histology

Mice were killed 8 weeks after the surgery. Knee joints were dissected, fixed in 4% paraformaldehyde for 24 h, decalcified in 10% EDTA for 2–3 weeks, paraffin-embedded, sectioned coronally at a thickness of 5 μm, stained with safranin O and fast-green, then stained with hematoxylin-eosin in accordance with the manufacturer’s instructions. OA severity was determined using the Osteoarthritis Research Society International (OARSI) scoring system (Glasson et al., 2010).

### Immunohistochemical analysis

Expression levels of CHOP, cleaved caspase-3, and SIRT1 were detected via immunohistochemical staining. Cartilage tissue was fixed with paraformaldehyde, embedded in paraffin, then cut into 5-mm-thick sections. The sections were then deparaffinized, treated with 3% hydrogen peroxide for 15 min, sealed with 5% normal serum, and blocked for 30 min. After incubation with primary antibodies against CHOP (1:300), cleaved caspase-3 (1:400), and SIRT1 (1:300) the sections were incubated with the secondary antibody. Images of the sections in each group were acquired via light microscopy. ImageJ version 6.0 was used to analyze each image. Levels of CHOP, cleaved caspase-3, and SIRT1 were determined via integral absorbance values.

### Statistical analysis

All data are representative of three independent experiments. All results are presented as mean ± standard deviation. All statistical analysis was performed using the unpaired Student’s *t*-test for two groups, or one-way analysis of variance for more than two groups, via GraphPad Prism version 9 (GraphPad Software, San Diego, CA, United States). *P* < 0.05 was considered statistically significant.

## Results

### Effects of melatonin on human primary chondrocyte viability with or without LPS

**FIGURE 1:**
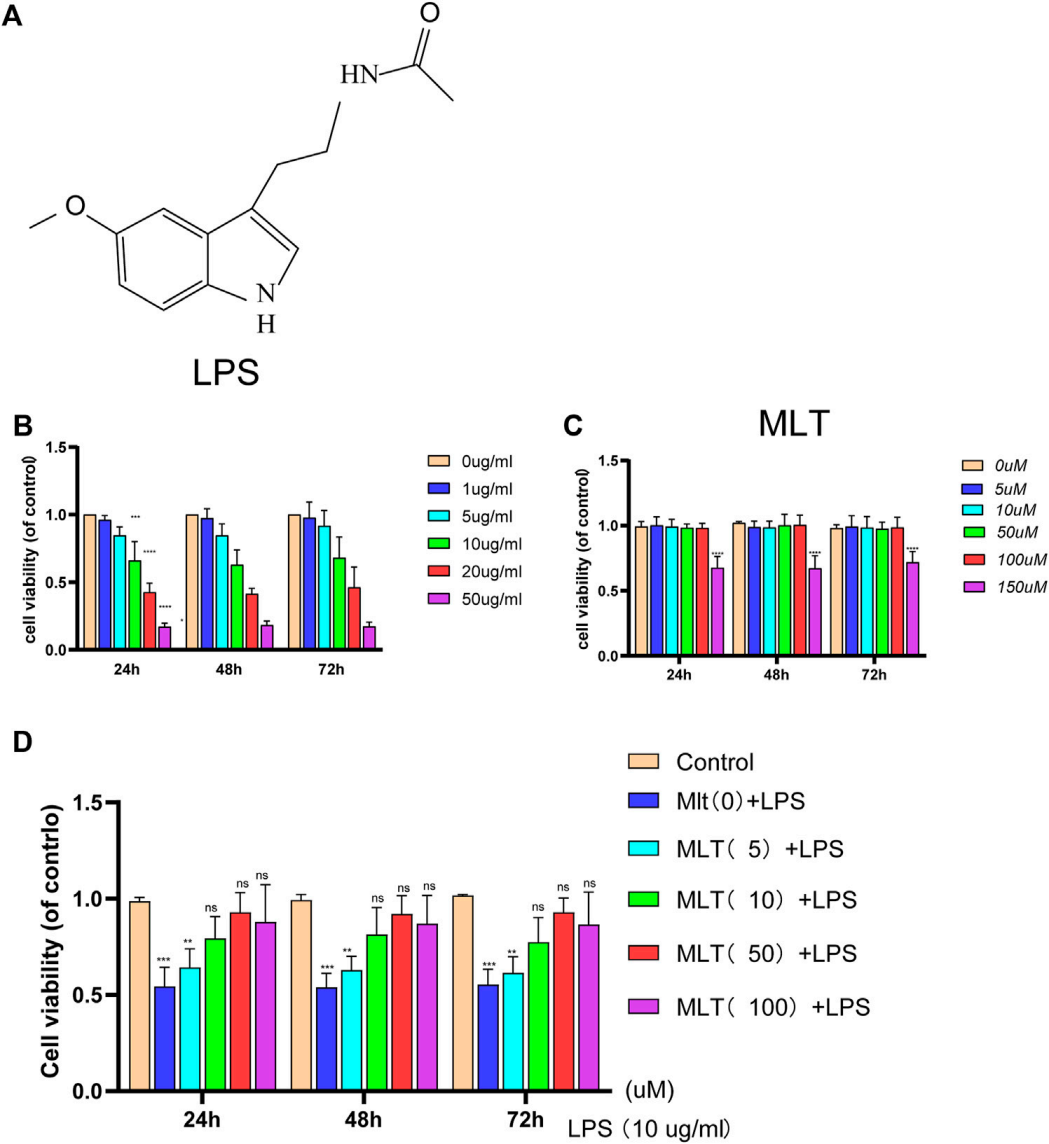
The effect of melatonin on the viability of human chondrocytes with or without lipopolysaccharide (LPS). **(A)** The chemical structure of melatonin. **(B,C)** The cytotoxicity effects of LPS and melatonin on human chondrocytes at different concentrations were tested at 24, 48, and 72 h using a cell–counting kit 8. **(D)** The viability of human chondrocytes treated with LPS (10 μg/ml) after different concentrations melatonin treatment. The experiment was repeated three times independently. All values are shown as mean ± standard deviation. **p < 0.01, ***p < 0.001, ****p < 0.0001. ns: not significant. *Abbreviations:* LPS, lipopolysaccharide; ns, not significant; MLT, melatonin.

CCK–8 assay was used to assess the effects of melatonin on chondrocyte viability at different time periods (24, 48, and 72 h) following treatment with or without LPS at different concentrations. An LPS concentration of 10 μg/ml obviously reduced chondrocyte viability (Figure 1B). Treatment with melatonin at a concentration of≤100 μM had no cytotoxic effect on chondrocytes (Figure 1C), and 10–100 μM of melatonin markedly alleviated LPS–induced cytotoxicity in a concentration-dependent manner (Figure 1D). Previous studies showed that a dose range of 10 nM–100 µM is ideal for studying the effects of melatonin (Liu et al., 2011; Quan et al., 2015; Xiong et al., 2015). Therefore, a dose of 10 μM melatonin was used as the therapeutic concentration. In addition, OA is a chronic degenerative joint disease characterized by persistent aseptic inflammation. Hence a relatively moderate amount of LPS (10 μg/ml) was used to stimulate human primary chondrocytes for 24 h to mimic aseptic inflammation in OA *in vitro*. Therefore 10 µM of melatonin and 10 μg/ml of LPS were used in the subsequent experiments.

### Melatonin protected human primary chondrocytes from LPS–induced apoptosis

**FIGURE 2:**
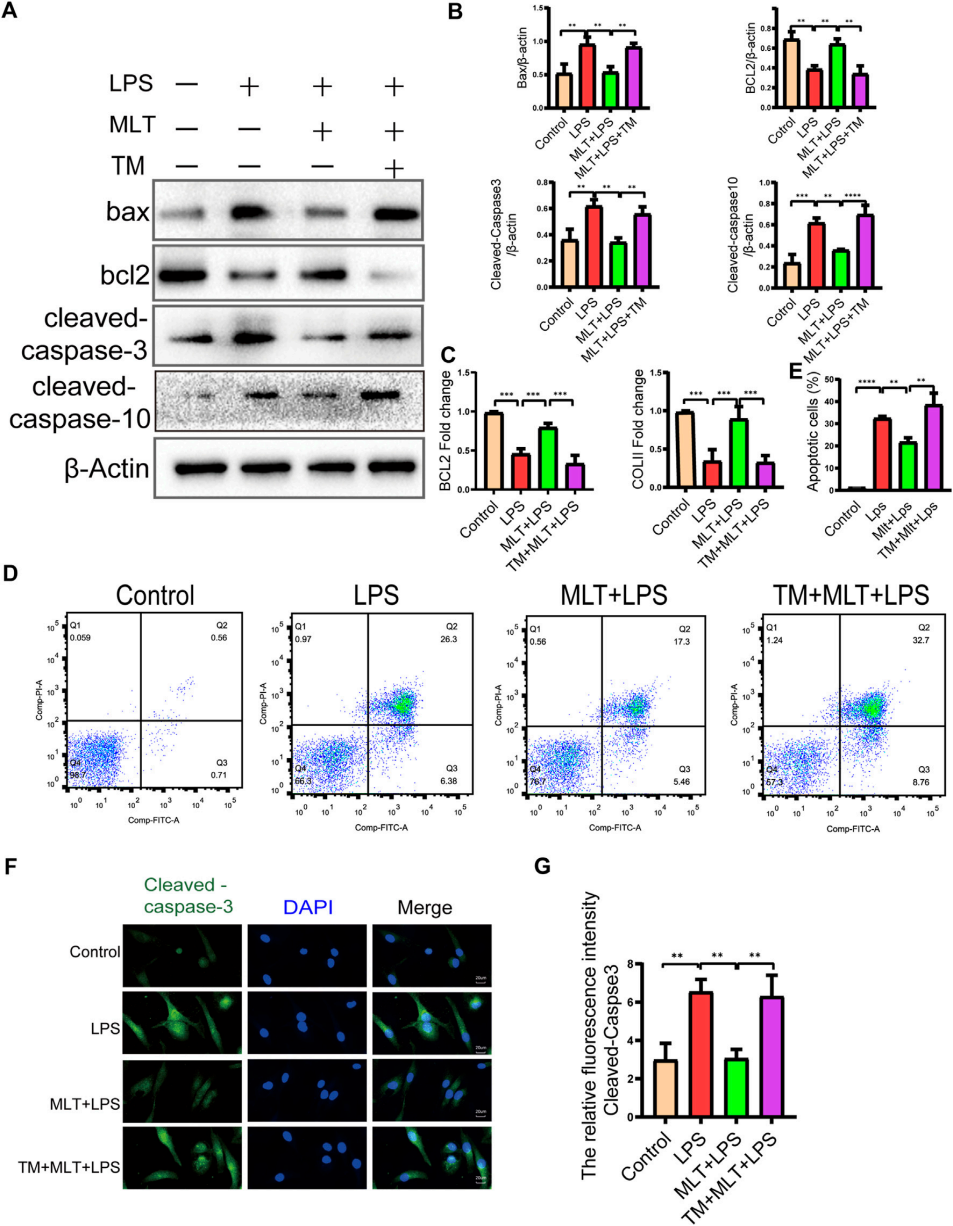
Melatonin inhibited apoptosis in human chondrocytes stimulated by lipopolysaccharide (LPS). **(A,B)** We detected the protein levels of Bcl–2, Bax cleaved caspase-10 and cleaved caspase–3 in each group. **(C)** mRNA expression levels of collagen II and Bcl–2 in each group were measured using qRT–PCR **(D–E)** Apoptosis of human chondrocytes were determined by flow cytometry following annexin V-PE and propidium iodide staining. **(F,G)** Quantification of the intensity of cleaved caspase–3 in human chondrocytes in each group was performed with immunofluorescence staining. The experiment was repeated three times independently. All values are shown as mean ± standard deviation. **p < 0.01, ***p < 0.001, ****p < 0.0001. *Abbreviations:* DAPI, 4′,6–diamidino–2–phenylindole; ns, not significant; PI, propidium iodide; LPS, lipopolysaccharide; MLT, melatonin; TM, tunicamycin.

To investigate the effects of melatonin on LPS–induced apoptosis in chondrocytes, we treated chondrocytes with 10 μg/ml of LPS with or without melatonin. First, primary human chondrocytes were pretreated with 10 μg/ml LPS for 2 h, and then with or without 10 μM melatonin for 24 h. Western blotting showed that melatonin suppressed pro–apoptotic proteins (cleaved caspase–3, cleaved caspase–10, Bax) and promoted a greater level of anti–apoptotic protein (bcl-2) compared to that in the LPS group (Figures 2A,B). The results revealed that the apoptosis of chondrocytes induced by LPS was significantly reduced after melatonin treatment. qRT–PCR results showed that melatonin–treated chondrocytes markedly elevated the expression levels of COLII and Bcl–2 compared to LPS–treated chondrocytes (Figure 2C), Flow cytometry and fluorescence analysis were used to assess the degree of apoptosis of chondrocytes (Figures 2D,E), and the results showed that LPS stimulation caused a significant increase in the apoptosis of human primary chondrocytes; however, the effect of LPS on apoptosis was significantly inhibited by melatonin. In addition, immunofluorescence staining for cleaved caspase–3 was consistent with the western blot results (Figures 2F,G). Therefore, the findings indicated that melatonin could inhibit LPS–induced chondrocyte apoptosis.

### Melatonin inhibits ERS in human chondrocytes induced by LPS

**FIGURE 3:**
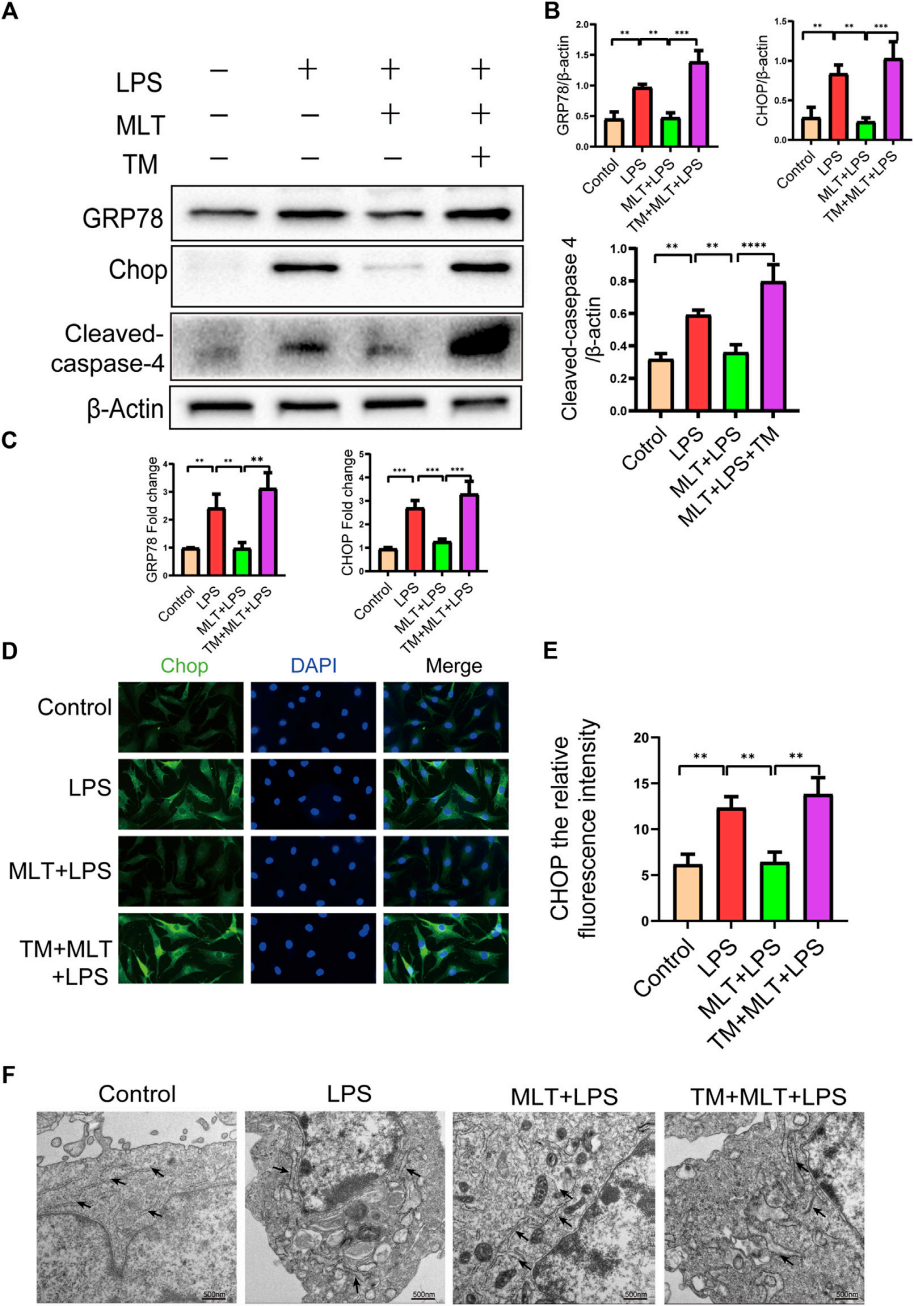
Melatonin inhibited inflammation–induced endoplasmic reticulum stress in human chondrocytes. **(A,B)** Western blot analysis Protein expression levels of GRP78, CHOP and cleaved caspas-4 were determined by western blotting. **(C)** The messenger RNA expression levels of glucose–regulated protein 78 (GRP78) and C/EBP homologous protein (CHOP) were checked by real–time polymerase chain reaction analysis in each group. **(D,E)** CHOP immunofluorescence staining. Obvious increasingly bright green spots indicate elevated CHOP expression (bar, 20 μm). **(F)** The effect of altered endoplasmic reticulum morphology after treatment was observed with transmission electron microscopy (bar, 500 nm) (black arrows refer to the endoplasmic reticulum). The experiment was repeated three times independently. All values are shown as mean ± standard deviation. **p < 0.01, ***p < 0.001, ****p < 0.0001. *Abbreviations:* LPS, lipopolysaccharide; ns, not significant; MLT, melatonin; TM, tunicamycin.

First, we evaluated whether the anti–apoptotic effect of melatonin inhibited ERS. The ERS–related biomarkers GRP78, CHOP and cleaved caspase–4 were analyzed by western blotting (Figures 3A,B). The messenger RNA expression levels of GRP78 and CHOP were analyzed by qRT–PCR (Figure 3C). The results showed that CHOP, GRP78 and cleaved caspase–4 were significantly increased in LPS–stimulated human primary chondrocytes. Moreover, treatment with melatonin could reverse the upregulation of ERS induced by LPS. Melatonin added to the human primary chondrocytes without LPS did not change the levels of CHOP, GRP78 and cleaved caspase–4 compared to those in the control group, indicating that Melatonin alone did not influence ER state of chondrocyte (figures no showed). The immunofluorescence staining for CHOP were consistent with the western blot and qRT-PCR results (Figures 3D,E). The results of transmission electron microscopy showed that the ER of the human chondrocytes treated with LPS was in an expanded state, and the expansion of the ER was reduced following treatment with melatonin (Figure 3F). ERS markers were also detected in human chondrocytes treated with TM, and TM attenuated the downregulation of ERS by melatonin.

### Melatonin attenuates LPS–Induced apoptosis of human primary chondrocytes by inhibiting ERS

To further explore whether melatonin inhibited ERS in human primary chondrocytes treated with LPS, we used TM, to activate ERS in human primary chondrocytes. Western blot results (Figures 3A,B) and qRT–PCR (Figure 3C) showed that the expression levels of CHOP, GRP78 and cleaved caspase–4 in human primary chondrocytes treated with TM were significantly increased compared to those in the melatonin + LPS group. In addition, the fluorescence staining of CHOP showed that TM increased the expression of ERS (Figures 3D,E). Transmission electron microscopy revealed the inhibitory effect of melatonin on ERS, while TM reverses the protective effect of melatonin on ERS (Figure 3F). We confirmed that melatonin protects chondrocytes from inflammation–induced apoptosis. To verify whether melatonin inhibited LPS–Induced chondrocyte apoptosis by inhibiting the ERS of human primary chondrocytes, we activated ERS using TM and measured the expression levels of pro–apoptotic biomarker, including cleaved caspase-3, cleaved caspase-10 and Bax and anti–apoptotic biomarkers: Bcl-2, (Figures 2A,B). Flow cytometry (Figures 2D,E) and immunofluorescence assays (Figures 2F,G) were also used to detect the level of apoptosis of human primary chondrocytes treated with TM. In summary, these results revealed that TM can reduce the anti–apoptotic activity of melatonin and melatonin attenuates apoptosis by inhibiting ER stress.

### Melatonin enhanced SIRT1 expression and inhibited the IRE1α-XBP1S-CHOP pathway in LPS-Treated human primary chondrocytes

**FIGURE 4:**
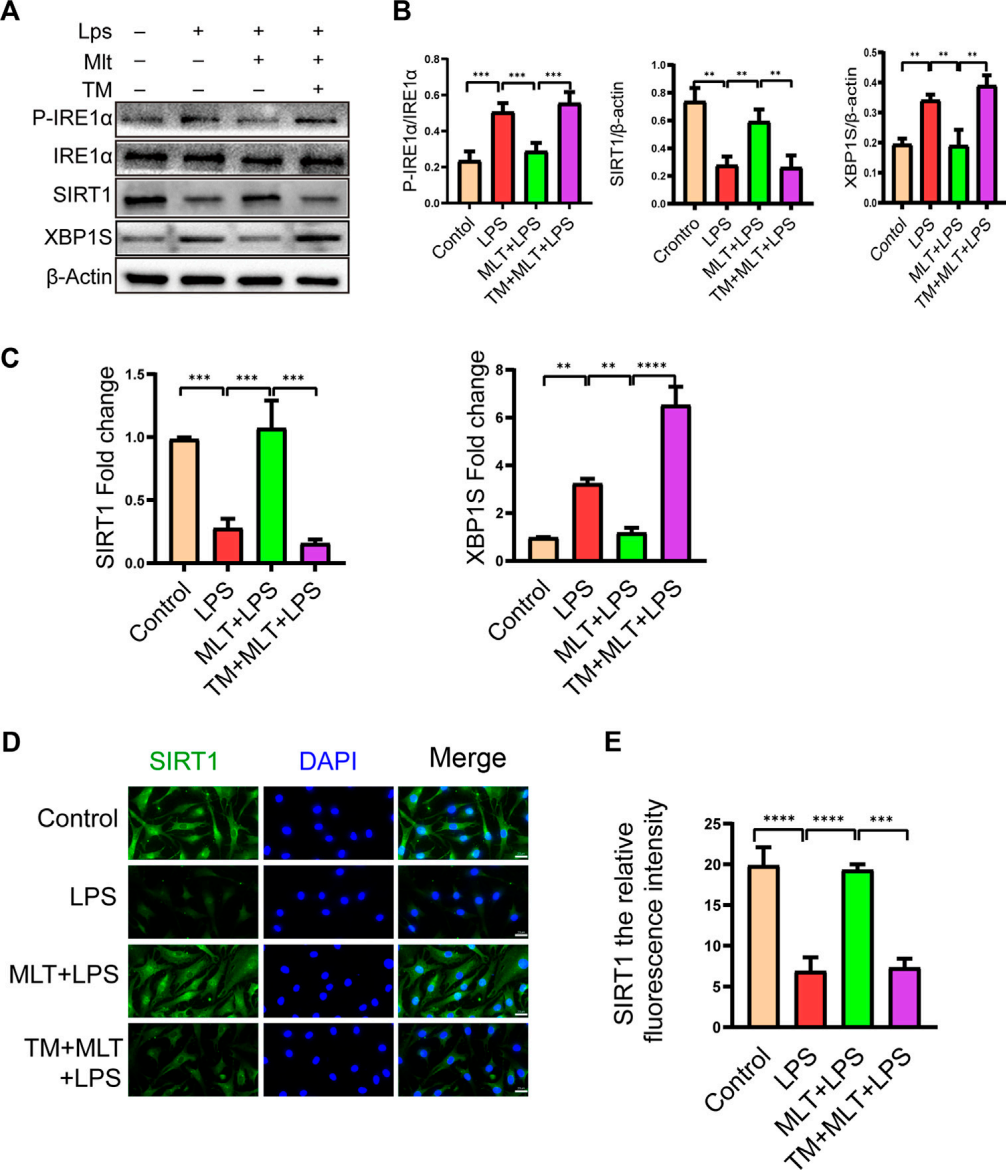
Melatonin upregulated the expression of sirtuin 1 (SIRT1) and attenuated the activation of the inositol–requiring enzyme 1α–X–box–binding protein 1 (IRE1α*–*XBP1S) pathway. **(A,B)** The protein expression levels of SIRT1 and *P*-IRE1α, *P*-IRE1α and XBP1S proteins were determined by western blot analysis. **(C)**We evaluated the messenger RNA expression level of SIRT1 and XBP1S by real–time polymerase chain reaction analysis. **(D,E)** SIRT1 immunofluorescence staining and quantification of the number of SIRT1–positive human chondrocytes in different groups. The experiment was repeated three times independently. Significantly increased green spots indicate that SIRT1 protein expression is upregulated (bar, 20 μm). All values are shown as the mean ± standard deviation. **p < 0.01, ***p < 0.001, ****p < 0.0001. *Abbreviation:* ns, not significant.

According to our results, melatonin increased the expression of SIRT1 and inhibited the IRE1α–XBP1S–CHOP pathway in chondrocytes treated with LPS. Previous research has shown that the expression of SIRT1 in OA chondrocytes is significantly lower than that in normal cells (Sacitharan et al., 2020). Therefore, we measured the expression levels of SIRT1 and IRE1α–XBP1S–CHOP pathway–associated proteins. As shown in Figure 4, LPS reduced SIRT1 expression in human primary chondrocytes, and melatonin treatment eliminated the inducing effect of LPS. We also observed that the SIRT1 protein expression level in chondrocytes treated with melatonin alone did not change compared to that in the control group. In addition, our results revealed that melatonin attenuated p–IRE1α and XBP1S protein levels in LPS–stimulated human primary chondrocytes (Figures 4A,B). The results of qRT–PCR (Figure 4C) and SIRT1 immunofluorescenc staining (Figures 4D,E) were consistent with the western blotting. Immunofluorescence results showed that SIRT1 can co-localize with IRE1α (Supplementary Figure S1), which provides a basis for the study of the interrelationship between SIRT1 and IRE1.

### Melatonin inhibits ERS–Related human primary chondrocyte apoptosis by promoting SIRT1 activation and inhibiting the IRE1α-XBP1S-CHOP signaling pathway in chondrocytes

**FIGURE 5:**
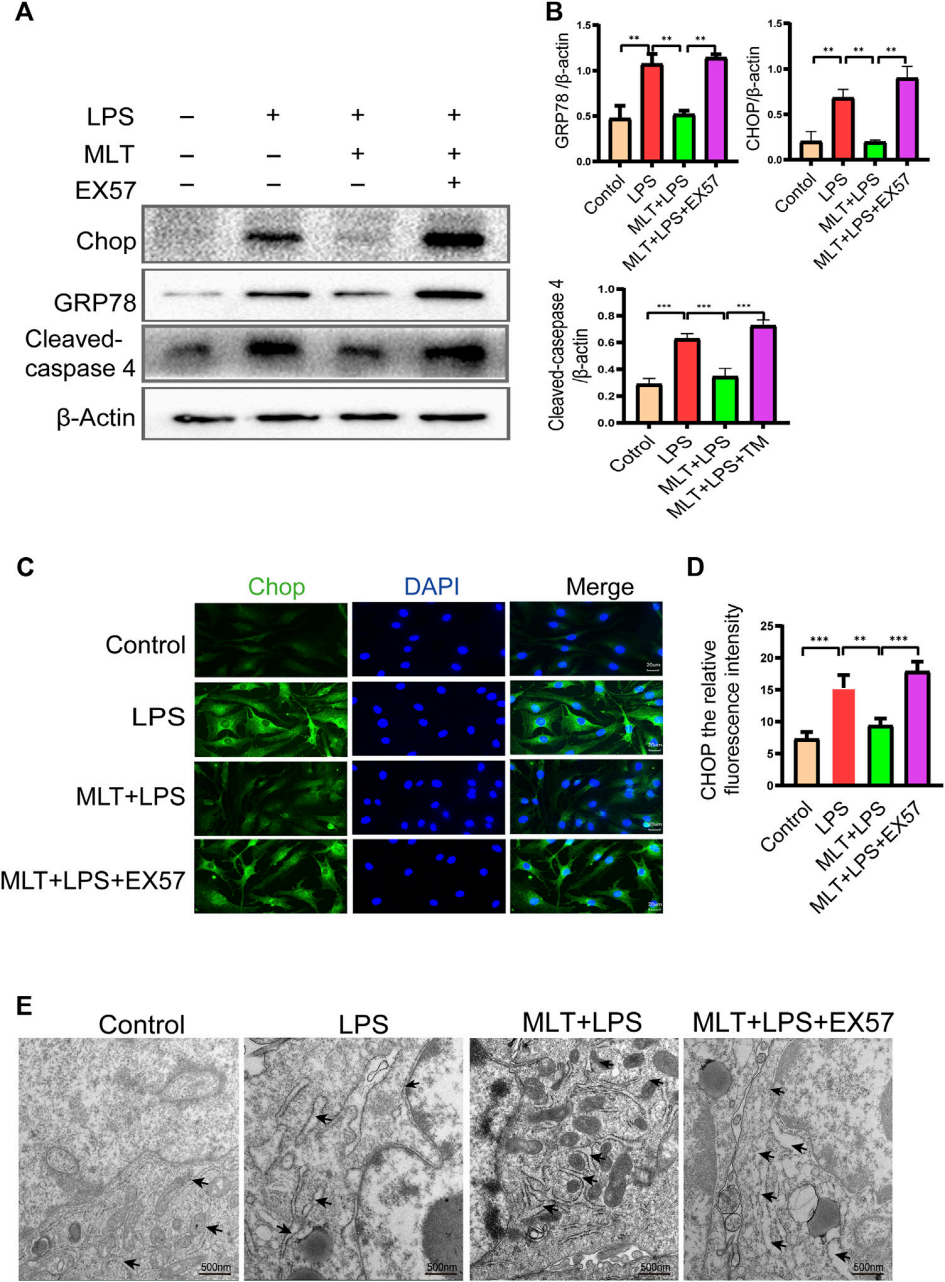
EX527 attenuated the inhibitory effect of melatonin on the endoplasmic reticulum stress of human chondrocytes induced by inflammation. **(A,B)** Expression levels of GRP78, C/EBP homologous protein (CHOP) and cleaved caspase-4 was determined by western blotting. **(C,D)** A representative CHOP level was detected by immunofluorescence staining to the endoplasmic reticulum. ImageJ was used to detect the fluorescence intensity of CHOP (bar, 20 μm). **(E)** The effect of altered endoplasmic reticulum morphology was observed with transmission electron microscopy (bar, 500 nm (black arrows refer to the endoplasmic reticulum); TM, tunicamycin; EX527, a classic sirtuin 1 inhibitor. The experiment was repeated three times independently. All values are shown as mean ± standard deviation. *p < 0.05, **p < 0.01, ***p < 0.001, ****p < 0.0001. *Abbreviations:* MLT, melatonin; ns, not significant.

To assess the roles of SIRT1 and IRE1α-XBP1S activation in ERS–Induced chondrocyte apoptosis, cells were treated with TM to activate ER stress. Our results revealed that TM downregulated SIRT1 expression and upregulated *P*–IRE1α and XBP1S expression (Figures 4A,B). In addition, we used EX527, a known SIRT1 inhibitor, to treat human chondrocytes. Our results showed that the protein levels of GRP78, CHOP and cleaved caspase-4 were elevated in the EX527 (10 μM) + melatonin + LPS group (Figures 5A,B). CHOP immunofluorescence result was similar to the western blot results (Figures 5C,D). In addition, transmission electron microscopy observed the extent of ER expansion of chondrocytes, and melatonin inhibited ERS (Figure 5E). These results demonstrated that inhibition of SIRT1 and activation of IRE1α are related to chondrocyte apoptosis induced by ERS.

### Melatonin inhibits the ERS of human primary chondrocytes induced by LPS by promoting SIRT1 expression and inhibiting the IRE1α-XBP1S-CHOP signaling axis

**FIGURE 6:**
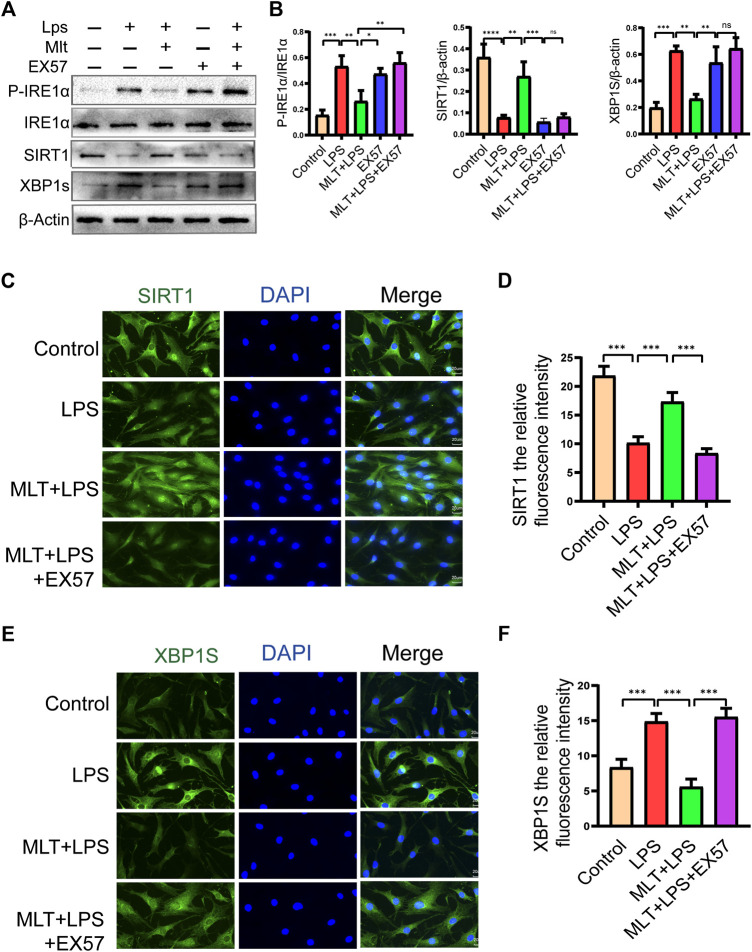
EX527 eliminated the protective effect of melatonin on human chondrocytes stimulated by lipopolysaccharide by inhibiting the inositol–requiring enzyme 1α–X–box–binding protein 1s (IRE1α–XBP1S) pathway. **(A,B)** Western blot analysis was performed to detect the protein expression level of SIRT1 and *P*-IRE1α, IRE1α and XBP1S after EX527 treatment. **(C–F)** Immunofluorescence staining of sirtuin 1 (SIRT1) and XBP1S (bar, 20 μm). ImageJ was used to detect the fluorescence intensity of SIRT1 and XBP1S. The experiment was repeated three times independently. All values are shown as the mean ± standard deviation. *p < 0.05, **p < 0.01, ***p < 0.001, ****p < 0.0001. *Abbreviations:* LPS: lipopolysaccharide; MLT, melatonin; ns, not significant; TM, tunicamycin; EX527, a classic SIRT1 inhibitor.

Melatonin inhibited LPS-induced apoptosis in human primary chondrocytes by promoting SIRT1 expression while suppressing the IRE1α-XBP1S-CHOP pathway. However, the interaction between SIRT1 and the IRE1α-XBP1S-CHOP pathway remains uncharacterized. To further investigate the connections between SIRT1 and IRE1α-XBP1S-CHOP, EX527 was used to downregulate SIRT1 expression in human primary chondrocytes. EX527 treatment reduced the levels of SIRT1 protein. P-IRE1α, XBP1S, and CHOP protein levels were reduced by melatonin, and P-IRE1α and XBP1S protein levels in chondrocytes treated with LPS were increased (Figures 6A,B). SIRT1 and XBP1S immunofluorescence results were consistent with the western blotting (Figures 6C–F). Collectively the data indicate that SIRT1 inhibits ERS in human primary chondrocytes by inhibiting the IRE1α-XBP1S-CHOP pathway.

### Melatonin attenuates mouse chondrocyte apoptosis, inhibits ERS, and delays OA progression in a mouse model

**FIGURE 7:**
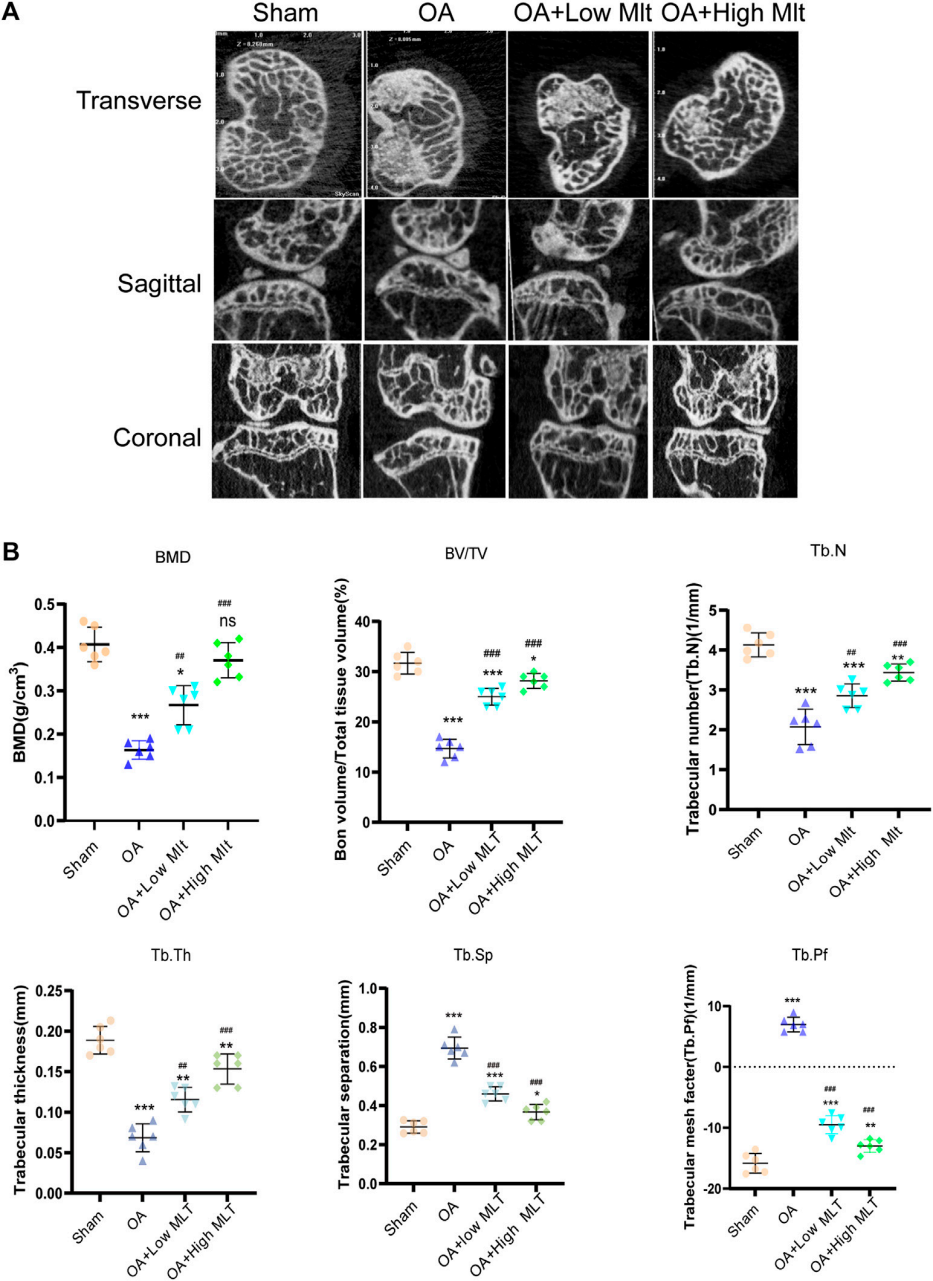
Preventive effect of melatonin on OA development in mice. **(A,B)** Analysis of bone destruction and knee joint bone histomorphometric parameters using micro -CT data, Data were shown as mean ± standard deviation and analyzed using one-way ANOVA (each group n = 6). *p < 0.05, **p < 0.01, ***p < 0.001, ns: not significant versus the normal group, #*p* < 0.05, ##*p* < 0.01, ###*p* < 0.001, ####*p* < 0.0001, ns: not significant versus the OA group.

**FIGURE 8:**
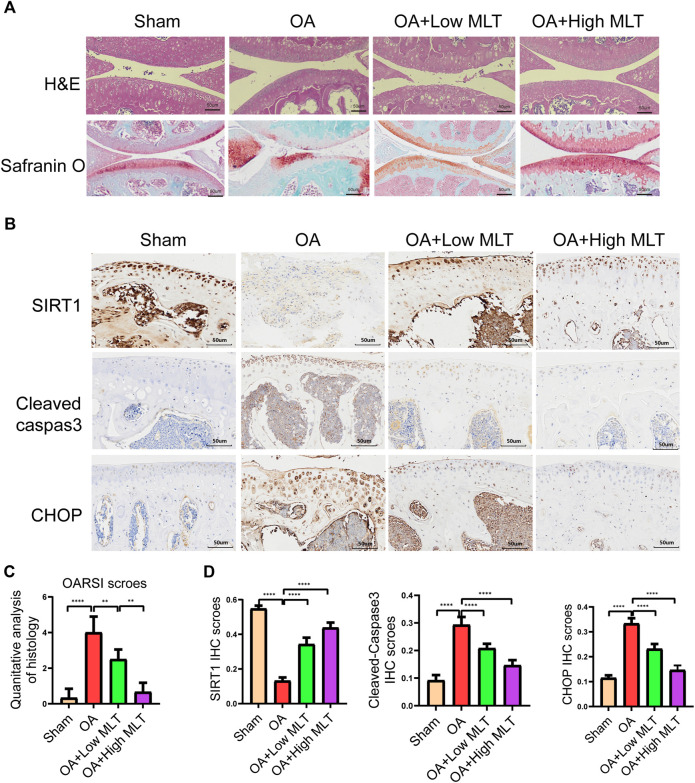
Melatonin inhibited apoptosis of chondrocytes and endoplasmic reticulum stress in the mouse ACLT model. **(A)** Hematoxylin and eosin staining safranin O staining and (bar, 50 μm) were performed to complete histological analysis and microscopic observation of cartilage destruction in each group at 8 weeks after surgery. The pathological manifestations of osteoarthritis were cartilage destruction and defects. **(B,D)** Immunohistochemical staining and quantification of immunohistochemical staining of cleaved caspase–3, chop, and sirtuin1 expression in each group (bar, 50 μm). **(C)** The scores for the four groups of articular cartilage were based on the International Association for Osteoarthritis Research system, as shown in the figure. All values are shown as the mean ± standard deviation (each group n = 6). *p < 0.05 **p < 0.01, ***p < 0 0.001 ****p < 0.0001, ns: not significant versus the normal group. *Abbreviations*; IHC, immunohistochemical; high melatonin, 150 mg/ml; low melatonin, 50 mg/ml.

Changes in bone mineral density and bone morphology were observed via micro-CT. The subchondral region of the tibia was used as the region of interest to determine the percentage of bone, bone volume/total tissue volume (BV/TV), trabecular number (Tb.N), trabecular thickness (Tb.Th), trabecular pattern factor (Tb.Pf), and trabecular separation (Tb.Sp). In the treatment groups BMD, BV/TV, Tb.N, and Tb.Th were increased compared with the OA group, whereas Tb. Sp and Tb. Pf were decreased. These results indicated that melatonin attenuated OA in a dose-dependent manner (Figures 7A,B). Melatonin reversed this situation in a concentration-dependent manner. Hematoxylin and eosin staining and safranine O of tissue from the knee joints of sham group mice revealed that the articular cartilage was smooth and red (Figure 8A). In the OA group severe damage, erosion, and destruction of articular cartilage were evident, but melatonin attenuated the apoptosis of chondrocytes and slowed the progression of OA in OA mice in a concentration-dependent manner. OARSI scores were highly consistent with the histology results (Figure 8C). OARSI scores in the OA group were markedly greater than those in the sham group, but melatonin reduced the OARSI scores in the OA group in a concentration-dependent manner. Immunohistochemical staining of mouse cartilage showed that melatonin attenuated the expression levels of cleaved caspase-3 and CHOP in OA cartilage, and increased SIRT1 expression compared to the OA group, and these results were similar to those of the *in vitro* studies (Figures 8B,D). Collectively these results indicate that melatonin inhibited ERS and had obvious anti-apoptotic effects in the *in vitro* and *in vivo* experiments.

## Discussion

OA is a multifactorial disease (Koyama et al., 2014). Despite numerous recent studies the pathogenesis of OA is not fully understood. Increasing evidence indicates that the onset of OA is related to the death of chondrocytes (Thomas et al., 2007; Ryu et al., 2012), and apoptosis of chondrocytes plays a crucial role in the onset and progression of OA (Dai et al., 2018; Park et al., 2020). The occurrence and development of OA are due to the loss of cartilage cells, resulting in the degradation of cartilage and thickening of subchondral bone. Mild inflammation and ERS are adaptive self-protection mechanisms of chondrocytes. Under prolonged or severe ERS conditions however, the dynamic balance of the ER cannot be restored, and the excessive activation of the UPR cannot be ameliorated (Xin et al., 2014). During unremittent ER stress chondrocytes activate the UPR, ultimately leading to apoptosis of cells (Tabas and Ron, 2011). Recent studies have shown that continuous inflammatory stimulation can lead to ERS (Zhang et al., 2019a), and the occurrence of ERS can initiate apoptotic signals through the IRE1α signaling pathway.

IRE1α is an ER transmembrane protein that plays a key role in regulating the UPR clock after ER stress activation. UPRs caused by long-term chronic inflammation can cause IRE1α to be phosphorylated, causing the splicing of XBP1, which results in the activation of downstream pro-apoptotic proteins including CHOP (Uehara et al., 2014). Notably IRE1α is involved in cell differentiation and extracellular matrix formation in chondrocytes, and regulates the expression of chondrocyte survival proteins including Bcl-2 and XBP1S (Han et al., 2013; Wu et al., 2018). In the current study the apoptosis biomarkers cleaved caspase-3 and Bax were upregulated in LPS-stimulated chondrocytes, whereas the anti-apoptotic protein Bcl-2 was downregulated. We proved during this study for the first time that melatonin significantly reduces the apoptosis of chondrocytes.

Typical biomarkers of ERS include CHOP and GRP78 (Malhotra and Kaufman, 2007; Uehara et al., 2014). In the present study the protein levels of CHOP, GRP78, and caspase-4 were significantly reduced by treatment with melatonin (10 µM). Moreover, melatonin inhibited ERS caused by inflammation. In mammalian cells, ER stress activates the three main ER-localized transmembrane signaling proteins IRE1α, PERK, and ATF6, which in turn activate UPR (Hotamisligil, 2010). Previous studies have shown that ERS promotes IRE1α phosphorylation, which leads to increased XBP1 cutting and promotes the degradation of OA cartilage (Walter et al., 2015). A delay in the development of OA was achieved by inhibiting ERS, thus, the excessive activation of IRE1α induced a terminal UPR that led to apoptotic signals via XBP1 messenger RNA splicing (Chen and Cubillos-Ruiz, 2021). We first found that melatonin relieved the ERS of chondrocytes by inhibiting the IRE1α-XBP1S-CHOP signaling pathway. The classic ERS inducer TM was used to further investigate the relationship between ERS and apoptosis in human primary chondrocytes. TM attenuated the anti-apoptotic effect of melatonin in chondrocytes. The above experimental results indicate that melatonin inhibited the ERS-mediated apoptosis of chondrocytes by inhibiting the IRE1α-XBP1S-CHOP signaling pathway, especially by directly inhibiting CHOP. However, whether the protective effect of melatonin on OA chondrocytes is achieved through the other two ERS-related pathways requires further experimental research. With the progression of OA, SIRT1 continues to decrease in the cartilage of OA patients. SIRT1 regulates expression of the extracellular matrix, and has anti-catabolic, anti-inflammatory, anti-oxidant, and anti-apoptotic effects (Deng et al., 2019). In chondrocytes SIRT1 can protect cartilage by regulating mitochondrial biogenesis, oxidative stress, autophagy, and ERS, and it can also inhibit chondrocyte hypertrophy and degeneration (Fujita et al., 2011; Wang et al., 2015). Therefore, SIRT1 has emerged as a promising therapeutic target for the treatment of OA.

Based on previous research results, the current study investigated whether melatonin inhibits inflammation mediated ERS and the apoptosis of chondrocytes through SIRT1. In this study, the experimental results indicated that LPS decreased the expression of SIRT1. Then, after treatment with melatonin, SIRT1 expression was significantly increased. To confirm that melatonin regulated ERS through SIRT1, we used an exclusive SIRT1 inhibitor, EX57, and we observed that EX57 significantly elevated the protein levels of CHOP, GRP78 and casepase-4, reversing the inhibitory effect of melatonin on the ER. These results indicate that SIRT1 inhibits the apoptosis of inflammation–induced chondrocytes by alleviating ERS. Previous study showed that SIRT1 plays a crucial role in regulating ERS–related apoptosis (Prola et al., 2017; Huang et al., 2018; Li et al., 2018). SIRT1 regulates the IRE1α–XBP1S–CHOP signaling pathway and promote the formation of growth cartilage plates (Chou et al., 2019). Previous research also demonstrated that SIRT1 activation can inhibit cadmium–induced ERS by inhibiting the IRE1α–XBP1S pathway (Romeo-Guitart et al., 2018). However, the relationship between SIRT1 and the IRE1α–XBP1S–CHOP signaling pathway in chondrocytes needs to be further explored. Human chondrocytes treated with the classic SIRT1 inhibitor EX57 showed markedly elevated expression levels of *P*–IRE1α, XBP1S, and CHOP. For the first time, our experiment has confirmed the importance of SIRT1 for ERS–mediated apoptosis and revealed that its mechanism of action is through the inhibition of the IRE1α–XBP1S–CHOP signaling pathway. Based on the above experimental results, we found for the first time that melatonin inhibits the IRE1α–XBP1S–CHOP signaling pathway by promoting SIRT1 expression in human primary chondrocytes induced by inflammation *in vitro*. However, whether the protective effect of melatonin and the expression of SIRT1 in OA are related to the other two pathways of ERS needs to be further verified. The typical features of OA progression are obvious destruction of articular surfaces and lesions and apoptosis of chondrocytes. The ACLT mouse model is a the widely used OA model in several studies (Zhou et al., 2019). Here, the mouse ACLT model was used to imitate the progression of OA. The concentration of melatonin varies greatly in animal models of different diseases. Zhang et al. (Zhang et al., 2019b)used a melatonin solution at a dose of 10 mg/ml in the treatment of ACLT models of OA, where intra–articular injection of the same amount (10 μL) of melatonin achieved a good curative effect. Meanwhile, other research has studied the effects of prenatal use of melatonin on the regulation of neonatal brain inflammation. In a study, intraperitoneal injection of melatonin (5 mg/kg) effectively reduced neonatal inflammation and related brain damage caused by maternal LPS (Carloni et al., 2016). According to another study, melatonin at a concentration of 10 mg/kg/day had a protective effect on COPD by reducing lung tissue cell apoptosis and ERS in COPD rats (He et al., 2019). Chen et al. (Chen S. J. et al., 2016) demonstrated that a melatonin dose of 200 mg/kg delivered through subcutaneous injection had a good effect on immune encephalomyelitis. In summary, melatonin at a dosage of up to 200 mg/kg is safe. In our experiments and related reports, the therapeutic effect of melatonin is dose–dependent, so we tested whether melatonin delayed the progression of OA at a low concentration of 50 mg/kg and a high concentration of 150 mg/kg. We assessed the effect of the drug on body weight in mice, these results show that melatonin attenuates weight gain with age in male mice (Supplementary Figure S2), similar to what has been reported in the literature (Tamura et al., 2021).

In the current study micro-CT indicated that melatonin could delay the progression of OA. We also found that in a mouse model of OA melatonin delayed joint degeneration and reduced chondrocyte apoptosis in a concentration-dependent manner. Our study revealed for the first time that melatonin inhibits the protein expression levels of CHOP and cleaved caspase-3 by promoting the expression of SIRT1. These results indicate that the potential mechanism of melatonin-based resistance to apoptosis is activation of SIRT1 expression, and inhibition of ERS.

**FIGURE 9:**
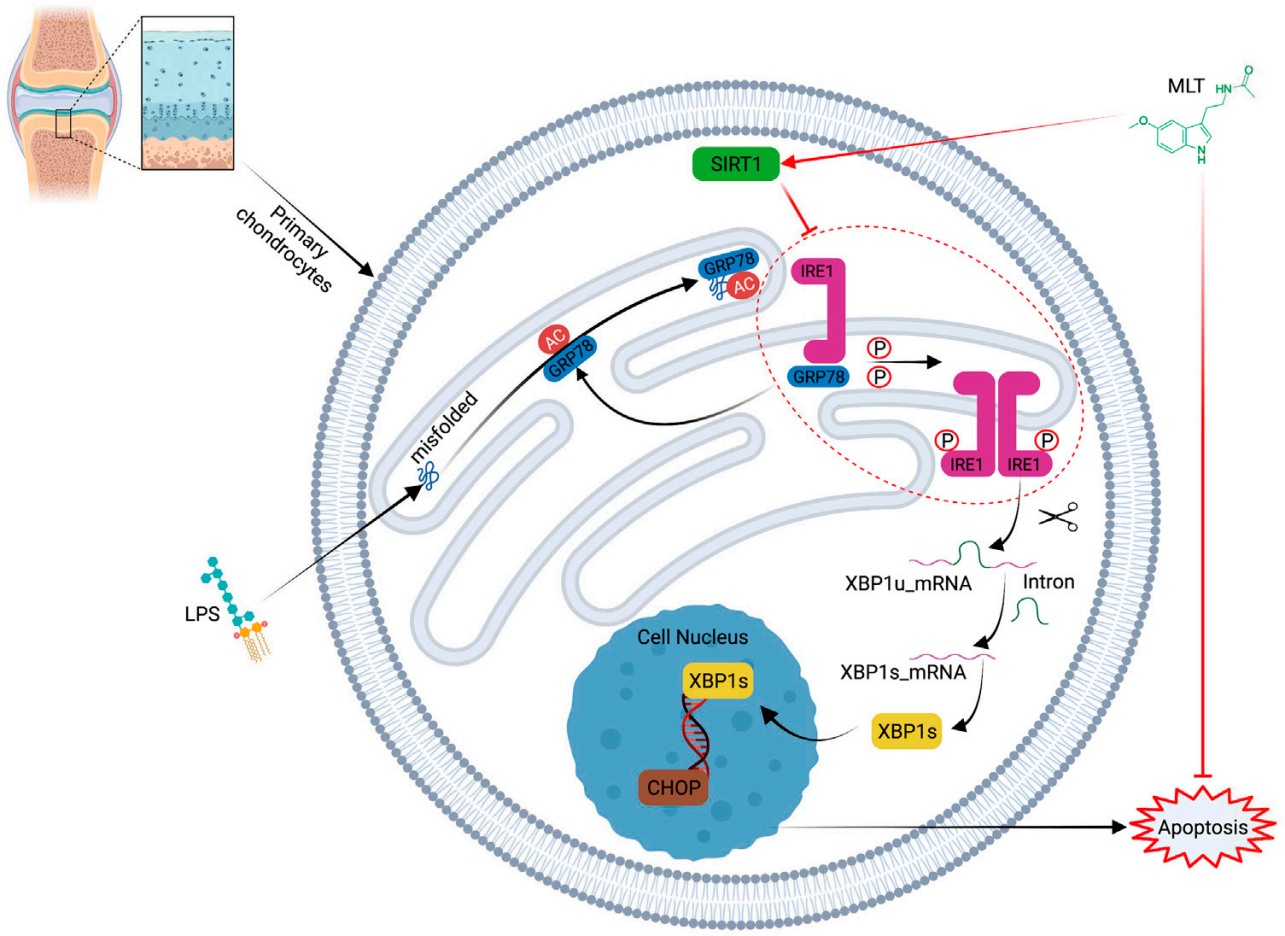
Schematic illustration of the mechanism of melatonin’s protective effects against lipopolysaccharide–treated human chondrocytes.

We conclude from our experiments that melatonin can delay the progression of OA by inhibiting ERS–induced apoptosis of chondrocytes *in vivo* and *in vitro.* Furthermore, melatonin protects articular cartilage by promoting SIRT1 expression and inhibiting ERS and apoptosis by blocking the IRE1α-XBP1S-CHOP signaling pathway (Figure 9). The protective effect of melatonin on cartilage was similarly verified *in vivo* experiments. Based on the above experimental results, melatonin may be a treatment option for OA. We also verified that melatonin reduces ERS of arthritic chondrocytes through the perk pathway (Supplementary Figure S3). Therefore, there results suggest that melatonin may alleviate chondrocyte apoptosis through the IRE1 pathway, but the IRE1 pathway is not the only pathway and further experimental exploration is needed regarding the effect of melatonin on ERS in chondrocytes.

## Data Availability

The original contributions presented in the study are included in the article/Supplementary Materials, further inquiries can be directed to the corresponding authors.

## Supplementary material

Supplementary Figure S1 The colocalization of SIRT1 with IRE1 by Immunofluorescence (bar, 20 μm).

Supplementary Figure S2 Effects of long-term melatonin treatment on body weight with aging. 10-weeks mice were divided into sham group, OA group, low–dose melatonin (50 mg/kg) and high–dose melatonin (150 mg/kg). Body weights were measured at 0, 2, 4 6, 8, 10, 12 weeks after operation, (each group n = 6).

Supplementary Figure S3 Melatonin attenuates the activation of PERK pathway
**(A)** Melatonin attenuated the phosphorylation of PERK. **(B)** EX527 eliminated the protective effect of melatonin on human chondrocytes stimulated by lipopolysaccharide by inhibiting the phosphorylation of PERK.
