# Supplementary figures and images for: Melatonin promotes sirtuin 1 expression and inhibits IRE1α–XBP1S–CHOP to reduce endoplasmic reticulum stress–mediated apoptosis in chondrocytes
Source: Front Pharmacol. 2022 Aug 11;13:940629. doi: 10.3389/fphar.2022.940629 (PMC9404507; doi:10.3389/fphar.2022.940629)

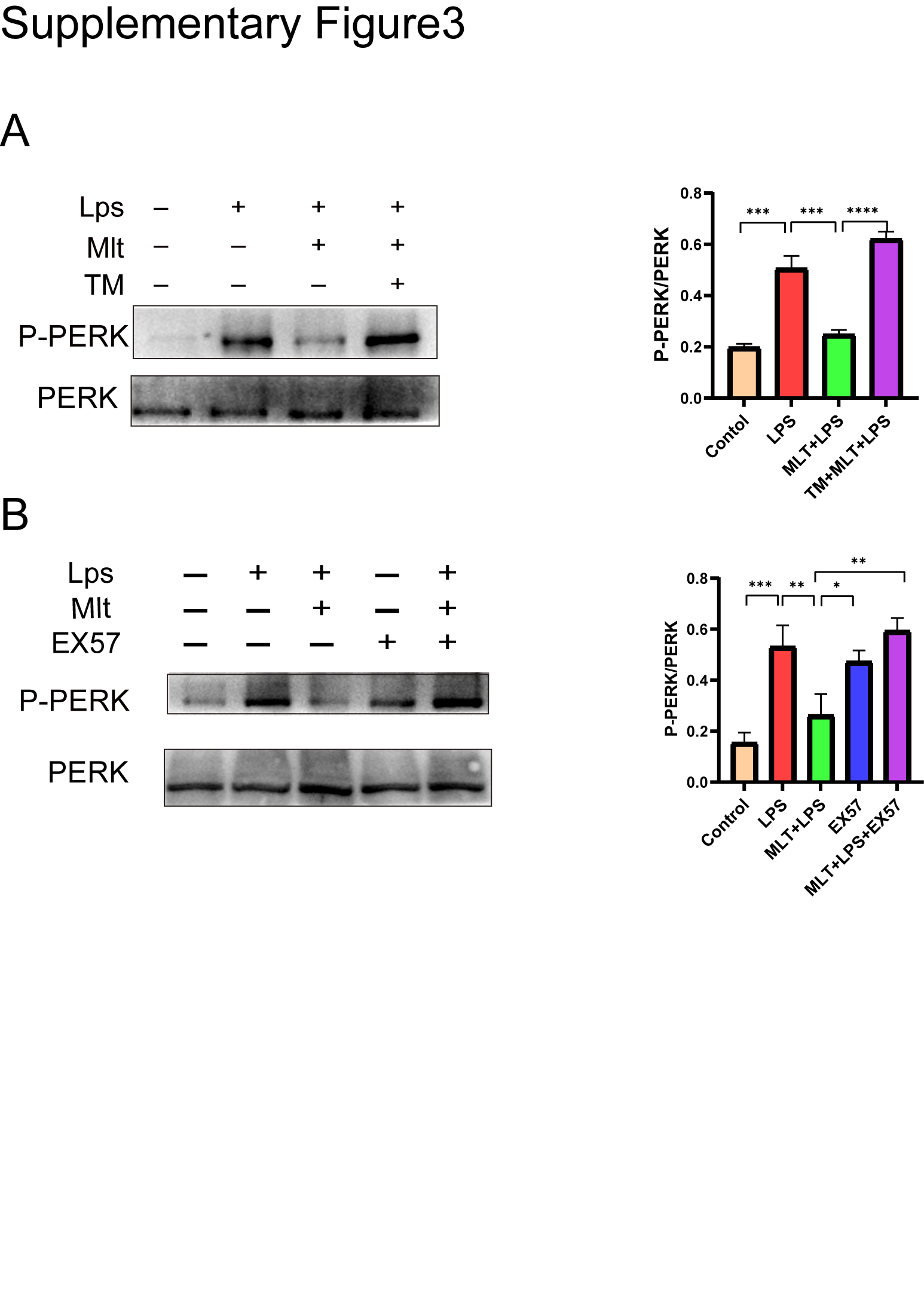

Supplement: Supplementary file 1 [file Image3.TIF]

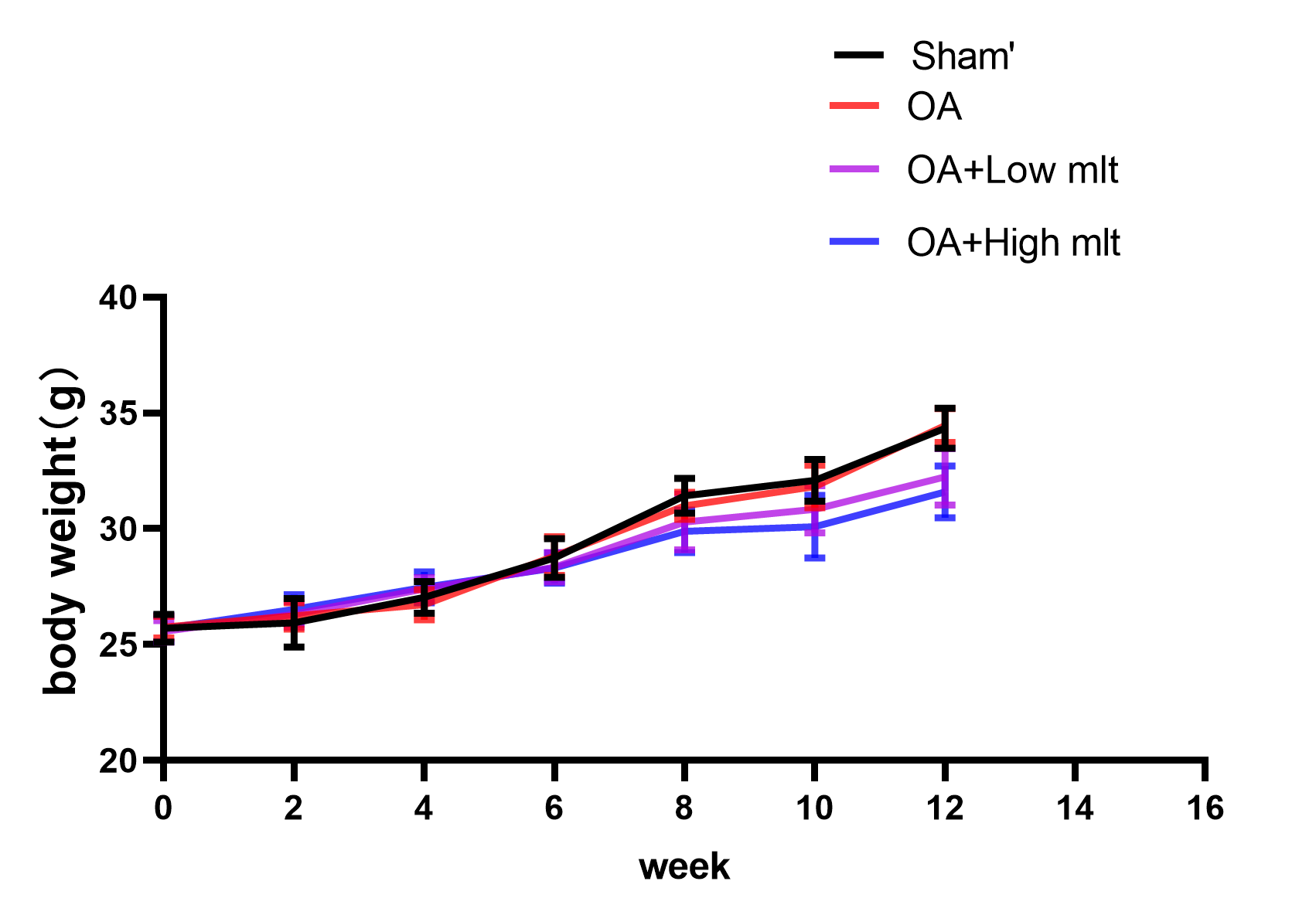

Supplement: Supplementary file 2 [file Image2.TIF]

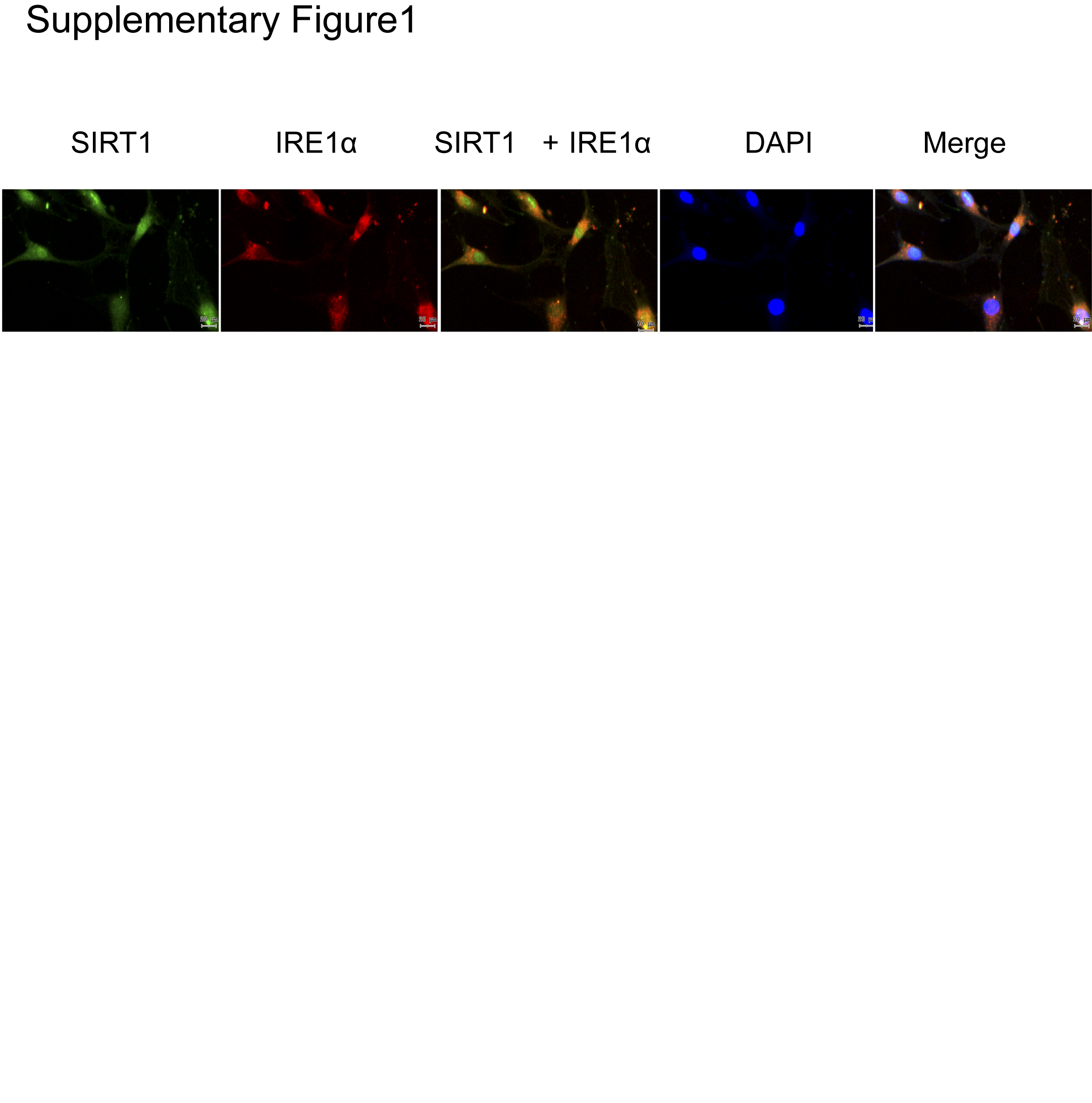

Supplement: Supplementary file 3 [file Image1.TIF]
